# Time, space and health: using the life history calendar methodology applied to mobility in a medical-humanitarian organisation

**DOI:** 10.1080/16549716.2022.2128281

**Published:** 2022-10-06

**Authors:** Juan-Carlos Cubides, Nuni Jorgensen, Paulo Cesar Peiter

**Affiliations:** a Parasitic Diseases Laboratory, Tropical Medicine Program, Oswaldo Cruz Institute, Oswaldo Cruz Foundation, Rio de Janeiro, Brazil; b Brazilian Medical Unit (BRAMU), Doctors Without Borders (MSF), Rio de Janeiro, Brazil; c School of Geography, Queen Mary University of London, London, UK

**Keywords:** Migration, information, methodology, humanitarian intervention, social determinants of health

## Abstract

In the medical humanitarian context, the challenging task of collecting health information from people on the move constitutes a key element to identifying critical health care needs and gaps. Médecins Sans Frontières (MSF), during its long history of working with migrants, refugees and mobile populations in different contexts, has acknowledged how crucial it is to generate detailed context-related data on migrant and refugee populations in order to adapt the response interventions to their needs and circumstances. In 2019, the Brazilian Medical Unit/MSF developed the Migration History Tool (MHT), an application based on the life history method which was created in close dialogue with field teams in order to respond to information needs emerging from medical operations in mobile populations. The tool was piloted in two different contexts: firstly, among mobile populations transiting and living in Beitbridge and Musina, at the Zimbabwe-South Africa border; and, secondly, among Venezuelan migrants and refugees in Colombia. This article describes the implementation of this innovative method for collecting quantitative retrospective data on mobility and health in the context of two humanitarian interventions. The results have proven the flexibility of the methodology, which generated detailed information on mobility trajectories and on the temporalities of migration in two different contexts. It also revealed how health outcomes are not only associated with the spatial dimensions of movement, but also with the temporalities of mobility trajectories.

## Background

Economic instability, conflict, political, religious and gender-based persecution, environmental change, humanitarian crisis and the ultimate search for work and better living conditions are all reasons, among many others, that compel people to leave their homes, thus shaping mobility dynamics in the world [1]. With an estimated 1 billion migrants and refugees globally [2], migration is now recognised both as a core social determinant of health and a global public health priority [3,4].

Migrant and refugees’ health outcomes tend to be negatively influenced by bureaucratic, economic and linguistic barriers to healthcare access; poor living and working conditions; and weak social support networks. Inequalities in the access to health services tend to become more acute over the course of migration processes, as migrants face discrimination and lack of information and are particularly vulnerable to infectious diseases and events of violence. Although the ‘healthy immigrant effect’ is well established in today’s global health literature, research has shown how immigrants’ health advantages tend to be gradually lost as mobile populations endure multiple forms of exclusion in host societies [5]. It is also important to consider how intersectionalities in terms of gender, age, ethnicity, and disabilities impact the health outcomes of mobile populations in different ways.

Although most prior research has focused on the health needs of migrants in destination countries, it is also important to discuss access to care in transit sites. As the result of border externalisation processes and lengthy asylum procedures, scholars have been calling attention to the growing complexity of migration trajectories. The different types of procedural hindrances created by destination countries to curb permanent settlement have generated at least two parallel processes: growing ranks of people who undertake long and dangerous journeys to seek asylum in high-income countries; and a large contingent of persons who must wait for unpredictable lengths of time in camps, or informal shelters, before having their claims judged or being resettled in a third country [6]. People in transit rarely have formal access to healthcare and are particularly vulnerable to violence and infectious diseases caused by their limited access to treated water and sanitation [7]. Exhausting journeys, long waiting times, and heightened uncertainty have also all been shown to be particularly detrimental to migrants and refugees’ mental health [8].

It is vital to acknowledge the particularities of cross-border mobility in terms of healthcare. This includes the pendular, seasonal and circular movement of people across national frontiers that do not necessarily fall within the category of migration. Cross-border mobility is often motivated by the search for healthcare (patient mobility), although it can also encompass traditional circulation patterns across neighbouring countries. These types of movement are particularly relevant in Africa and Latin America, where national boundaries have divided territories occupied by the same ethnic group. Although cross-border mobility is often a constitutive part of these societies, it also often poses unique challenges to states in terms of surveillance, planning and organisation of healthcare services [9].

## Humanitarian context, migration, and data

As a medical-humanitarian organisation working with mobile populations in different contexts, Médecins Sans Frontières (MSF) relies heavily on the available health data to design evidence-based interventions, yet detailed information on mobility trajectories and access to health care are not always readily available, particularly in low and middle-income countries [4,10]. To design, monitor and evaluate projects focused on mobile populations, MSF often needs retrospective health data in settings such as refugee camps and shelters, crossing points, detention, and reception centres. Even though it collects patient data for monitoring purposes in each of its projects, these are often not sufficient, as they do not include people who, for different reasons, have not reached assistance. To counter this problem, different partner sections and field projects within MSF also conduct cross-sectional population surveys and qualitative studies according to operational needs. Nonetheless, traditional questionnaires have often been reported as being too rigid to capture the complexities of mobility trajectories and how these are related to health outcomes in sufficient detail, as well as being subject to memory errors.

## Objective

To circumvent the limitations of traditional questionnaires, in 2019, the Brazilian Medical Unit/MSF piloted the Migration History Tool (MHT), an application based on the Life History Calendar method, which was tailored to MSF projects focused on mobile populations and their specific information needs. This paper aims to describe how the method and software have been implemented in two different settings and how it has contributed to the design of evidence-based medical humanitarian interventions.

## Methods

### The life history calendar

The MHT was based on the Life History Calendar methodology (LHC), the primary purpose of which is to gather high-quality, detailed biographic information. Traditionally, migration histories in quantitative research are usually collected through one or two simple questions, such as: ‘Where did you live in 2006?’ and “Where did you live before being in your current place of residence?’. However, in using these questions alone, researchers would miss all the mobility steps that occurred between the chosen date threshold and the last place of residence, as well as what happened prior to that date. When dealing with highly mobile populations, this could represent a significant loss of information.

While looking for a quantitative methodology ‘as flexible as migration itself’, the literature review process led researchers towards the Life History Calendar (LHC) methodology. The LHC aims to gather information on an individual’s previous places of residence in a visually optimised table format. In the model proposed by Carling in 2012 [11], the LHC is a grid in which the columns represent time periods, and the rows contain information on the places of residence lived in each of these periods, as well as key life events. Public events may also be recorded to help participants remember what happened in their life during a specific period of time. Thus, calendar methods have been conceived not only as a way of gathering detailed retrospective data, but also as a means of enabling a transversal analysis of the interviewee’s multiple life dimensions, and as a technique for improving biographical recall and reducing memory errors and bias. Research on the structure of autobiographical memory has revealed that traditional surveys generally fail to generate reliable data based on past events. In contrast, calendar-based methods are based on the principle that certain events work as cues for others. This means that events which happened across different domains, such as work, family, or public life, may be used to help the participant to recall others (parallel cueing), or they can be used sequentially, for example when a person is asked what move succeed or preceded the other (sequential cueing) [11].

### Migration history tool: the format

Whilst the LHC methodology can be readily used to generate and analyse biographical narratives of mobility from a micro-scalar, qualitative perspective, the use of this method in a quantitative fashion stumbles upon the challenge of collecting and processing data on the detailed migration trajectories of hundreds or even thousands of people. Based on the main aspects of the LHC, the MSF team therefore created a new quantitative data collection tool structured around the collection of time and location data, which could be linked with health information. As it has been named, the Migration History Tool, aimed to generate information on three dimensions of a person’s history: their lifetime migration experiences, the most recent migration experience (focused on migration routes), and plans and destination. The questionnaire corresponds then to a series of subordinate tables used according to each participant’s migration and life experiences. Following an assessment of the available survey applications that could be used to design the questionnaire, the team decided to develop a unique software, which could be specifically tailored to the LHC methodology. ^1^^1^The software was created with the sponsorship of MSF’s Transformational Investment Capacity.

The questionnaires inserted into the program comprised several modules on health issues, such as sexual and reproductive health, chronic conditions, infectious diseases, exposure to violence, and mental health. These modules are linked to the person’s past migratory experiences, current migratory experiences and future plans. The surveyed individuals were asked to report all the places where they had lived for more than three months (Global Migration) and, in some cases, all the places they had passed through in the most recent international journey (Current Migration). This data was inputted into a table, allowing researchers to capture not only people’s places of first and last residence, but the internal migration processes preceding their international migration, as well as return and remigration patterns. All migration-specific questions comprised a georeferenced component, which automatically pinpointed places on a map and generated specific geographic coordinates that could later be analysed in the form of maps or geostatistical models.

One of the main advantages of the software developed by MSF in relation to other applications or paper-based alternatives is that the grid format allows researchers to add questions related to each of the columns, which, in turn, could be linked to a specific place of residence or point of transit. So, for example, when reporting where they lived in 2007, participants were asked who they lived with and why they had moved to that specific place. For each subsequent place reported, the same questions were asked so that the instrument was standardised across all interviews. Unlike paper-based alternatives, the program allowed interviewers to add an unlimited number of columns, depending on each person’s history. Given the amount of data generated through this method, the grid-type questions tended to be short and focused solely on capturing participants’ mobility trajectories. The interview was then coupled with traditional multiple-choice or open-ended questions. Often, traditional questions were followed by date questions, which, in the analysis phase, enabled researchers to link specific health events to the data gathered through the grid format.

Notably, the grid questions could be tailored in accordance with the population studied. If this is a highly mobile population, it is not always necessary to know all the places the participants had lived in since birth, and date thresholds could be defined by researchers in the study design stage. Similarly, the criteria for the inclusion of places can also be defined in relation to the study’s objectives. For example, when studying migrants, the Global Migration module was generally focused on the places where the person had lived for at least three months. In contrast, for mobile populations who were not necessarily migrants, the calendar format could focus on which places a person had been in the past month (or in any chosen timeframe), and the approximate date when they had travelled to the destination. The Current Migration module followed a similar logic, and it was generally centred on the sites the migrant had passed through since the beginning of their journey. Additional features of the software include audio pre-recorded questions for gathering sensitive information.

### Field implementations

The tool was developed in close dialogue with field teams, to ensure that it responded to the information needs emerging from operations. Once the software application was finalised, the tool was piloted in two different contexts. The first setting chosen to conduct a mobility survey using the MHT was a project spanning Zimbabwe and South Africa, which focused on providing primary healthcare to mobile populations in transit, or who had recently arrived in Beitbridge and Musina, border cities in the Limpopo region. In sequence, a second survey using the same methodology was conducted in Colombia focused on Venezuelan migrants and refugees. The rationale behind the choice of such different research settings was to evaluate the adaptability of the method and software in relation to various information needs, and to assess the extent to which the tool was aligned with local ethical and cultural requirements.

The Beitbridge-Musina border is currently one of the busiest in the Southern Africa region, receiving daily groups of migrants from Zimbabwe, Malawi, Zambia, the Democratic Republic of Congo (DRC), Burundi and more distant countries, such as Pakistan, Somalia and Ethiopia. MSF activities in the region explicitly addressing displaced populations date back to the year 2007 and have included the provision of antiretroviral treatment (ART) and tuberculosis (TB) treatment to remote rural populations in South African farms and, more recently, the delivery of primary healthcare to Zimbabwean returnees from South Africa and mobile populations across the Limpopo River.

Although the Limpopo area is a very well-known context for MSF, information on migrants’ needs in the region is limited. The available IOM data was neither updated nor comprehensive in terms of health demands. The mobility context at the Beitbridge-Musina border is highly complex, as it comprises groups of different nationalities with distinct migration trajectories. While some migrants, refugees, and asylum seekers hoped to settle permanently in South Africa, groups of Zimbabwean small farmers and farmworkers also crossed the border daily in search of work, products, and medical help. In addition, the project also encompassed Zimbabwean returnees who had previously been detained in South Africa, and who presented particular healthcare needs. Considering this diversity, the research team decided to separate the study into five different modules, each of which was centred on a distinct target population. Through the use of the Migration History Tool, a total of 1.735 people were interviewed in several catchment areas in the region from January to March 2020. The survey instruments were fully translated into Shona, Venda, Ndebele, and Swahili, and the interviews were conducted in the language which the respondent felt most comfortable with. This was the first known survey to describe the socio-demographic profile, health-seeking behaviour, and migratory trajectories of migrants and refugees in the Limpopo area.

In the case of Colombia, the stark increase in the number of Venezuelan migrants and refugees in recent years constitutes an unprecedented situation not only for the country but in Latin America (UNHCR-R4 V). The migratory dynamics in Venezuela have undergone a huge and markedly observable transformation over the last five years, from being a country that historically attracted migrants (especially from neighbouring Colombia) to one that in just three years (from 2016 to 2018) reached a negative balance in emigration terms [12]. Various estimates converge to indicate that Colombia leads the group of recipient countries in the region, hosting approximately 32% of the total Venezuelan migrants and refugees, followed by Peru, Chile, and Ecuador [13].

There is still great difficulty in establishing the precise numbers and specific profiles and needs of the Venezuelan migrants and refugees crossing borders mainly due to the large number of non-official crossing points [14]. Additionally, Colombia and Venezuela share a long history of population exchanges in a particularly fickle socio-political context where habitually, the population living in frontier areas exhibit patterns of circularity and constant mobility along a highly permeable border, with a large number of people mobilising in search of employment, supplies, and access to basic health care and education services [15].

In 2018, MSF established basic healthcare projects in three of Colombia’s border states (Guajira, Norte de Santander, and Arauca). MSF’s activities with Venezuelan migrant and refugees’ populations cover two work fronts: firstly, institutionalised medical activities, in-hospital spaces where primary health care services are provided (general medical consultation, sexual and reproductive health, mental health, and prenatal care, among others) and secondly, with mobile teams providing the same services described above, adding points of care for long-distance walkers. MSF activities in the border areas were established as a starting point upon which explore the distribution and conditions of the Venezuelan population in the territory and to refine a research analysis adapted to projects’’ information needs. The overall research activity had 516 Venezuelan migrants and refugees participants.

It is important to highlight that in the implementation of the survey in the three countries, the municipalities and specific sites chosen corresponded to a selection based on MSF’s activities and operational criteria. Once the locations were chosen, independent, statistically significant samples were then drawn as representative of those specific populations. Thus, the results of this research activity cannot be applied to migrants and refugees on a national scale. Also, the original scope of the project was reduced due to COVID contingency causing the loss of qualitative data that had been intended to be collected to help the interpretation of findings.

## MHT implementation outcomes and lessons learnt

As states’ strategies of deterrence multiply, scholars have started to look deeply into the temporal dimension of migration beyond its more commonly acknowledged spatial dynamics [6]. The times of migration are clearly interconnected with health, as these spaces of transit and waiting are often marked by the discontinuation of care, the onset of illnesses that cannot be diagnosed or treated, increased vulnerability to physical violence, and a general lack of care that may have severe consequences for migrants’ mental health.

In the case of Musina (South Africa), one of the main added values of the implementation of the MHT has been the estimation of the length of time migrants and asylum seekers stay in the city. The research at this site revealed that mobile populations remain, on average, for nine months in Musina, with the duration of stay rising to 16 months among Zimbabwean women. The reasons for staying for so long in a place that is often considered to be a transit point varied by nationality, with Zimbabweans more often giving work-related reasons, whilst Congolese generally pointed out the lengthiness of asylum application procedures. By linking up the residence times with the times of meaningful events, the survey revealed that protracted periods of residence in the city were associated with greater exposure to violence, particularly sexual violence. It also revealed that this association was stronger among men living in the city’s shelter, which indicated the need for the organisation to strengthen programs for this demographic, including the design of evidence-based advocacy strategies.

Time has also been a key variable in exploring the trajectories of detention among Zimbabwean migrants in South Africa. Following the sequential queuing technique enabled by the tool, the MSF team asked every person who arrived at the Beitbridge Reception Centre in Zimbabwe about the places they had been sent to after their arrest in South Africa, which included detention centres, police cells and prisons. Contrary to common sense, we found that detention pathways in the country were far from straightforward. The respondents had been to 2.6 facilities on average, with some passing through six different places, and 25% of them had spent more than three and a half years incarcerated before being deported. These figures represent a serious violation of the maximum length of time an unauthorised migrant can rightfully be detained in South Africa − 120 days. The findings showed that conditions in police stations were poorer when compared to those in Lindela, and that migrants sent to police stations rarely had access to health care, including continuous care for chronic conditions. Whilst most advocacy in relation to the detention of migrants in South Africa has been focused on large centres, such as Lindela, the survey revealed the need to also look at much less visible settings, particularly police stations.

There are important aspects to highlight too. The survey conducted in South Africa evidenced the differences in migration trajectories of migrants of various nationalities. Whilst living conditions in South Africa were similar across all the groups analysed, Burundians had been in transit for longer periods of time, which also increased their chances of having been exposed to violence in the years preceding the survey. Operationally, the identification of points of transit, border crossing sites, and places of habitual residence through the study helped the organisation to pinpoint locations where further needs assessments could be conducted, whether in South Africa, Zimbabwe or third countries.

Also, in relation to the Colombian experience, the implementation of the MHT methodology allowed the exploration of mobility dynamics among Venezuelan migrants and refugees in a highly volatile environment. The analysis of the data collected from the Venezuelan population interviewed population evidenced a migration profile characterized mainly by family groups with more extended permanence plans, who were searching for better living conditions, access to sources of employment, education, and health services. This group migration characterisation reflects that more than half of the influx were young people under 20 years old, with a striking 20% of children aged under five years old. Therefore, MSF operations in Colombia, working with Venezuelan migrants, are then called to shape solutions to guarantee longer-term access to health services for these populations.

On average, interviewees reported having lived for more than three months in between one and six different cities, towns, or villages, with an average of 1.6 locations per respondent. The calculated mean time of stay in the current residence was four months, revealing that Venezuelan migrant populations at the interview sites have a low mobility profile and a tendency for long-term stay. In addition, when analysing the routes per country, it is interesting to note in the recent migration process that 91% of the travellers did not make a stop inside Venezuela. This is most probably related to the fact that most interviewees were previously residing in bordering states. For those who did stop along the route inside Venezuela, the maximum number of places reported was two. In contrast, a wider mobility pattern can be identified when analysing Colombian locations, where people had made travel travel stops in up to ten different places, many of them in the interior of the country, with a return trip to settle down later in one of the border cities where they were interviewed. This information indicated the importance of evaluating the provision of health care in border municipalities.

In terms of access to health care, the survey found that, among people who searched for health care in their place of residence, 27% received assistance in less than one hour; 37% were assisted between one and three hours after seeking the service and 21% were assisted three or more hours after requesting help, but within the same day of searching for services. However, when long-term care is considered, the survey found that 49% of the people afflicted by chronic diseases were out of treatment, with a set average time of not having received medication of approximately 3.6 years at the time of the interview. The main reasons given by respondents to explain their treatment discontinuation included not having the money to buy the treatment (30%) and not having access to a proper health service (15%). This data confirmed need to establish a comprehensive package of care for Venezuelan migrants and refugees that would ensure not only emergency attention (as is intended by the Colombian health system), but that guarantees the long-term provision of care.

Finally, it is important to highlight that the use of the Migration History Tool has generated intense debate within MSF regarding the ethical implications of the information generated, and to the data collection process itself. The main concern was that the documentation and dissemination of detailed migration routes could be used by national states to stiffen their border controls at crossing points. To counter this possibility, several data protection measures have been adopted, including restricting the results that would be published and shared externally. There was also apprehension that the in-depth exploration of life trajectories – including events of violence – could lead to the re-traumatisation of people included in the study. To minimise the risk to participants, some very sensitive questions were not included in the research. In addition, data collection teams were accompanied at all times by mental health staff.

## Conclusion

This article has sought to describe the application of a novel software, based on the life-history calendar methodology, in the collection of quantitative retrospective data on mobility and health in the context of two humanitarian interventions. Particularly, it has revealed how health outcomes are not only closely linked with the spatial dimensions of movement, but also with the temporalities of mobility trajectories. Whilst taking key ethical considerations into account, the information generated through this method could enable organisations working with mobile populations, in health-related activities and beyond, to better define their scope of action, target demographics, attention sites and advocacy strategies.

## Paper context

Availability of specific information on migrants and refugees’ health conditions and access to care is essential to determine critical health gaps and adapt response interventions to their needs and circumstances. The highly volatile trait of the migration phenomenon makes particularly relevant a consistent context analysis and information flow. For this reason, Doctors Without Borders (MSF) has implemented the Migration History Tool (MHT) as an innovative methodology for collecting information from people on the move.
